# Simultaneous analysis of the of levamisole with triclabendazole in pharmaceuticals through developing TLC and HPLC–PDA chromatographic techniques and their greenness assessment using GAPI and AGREE methods

**DOI:** 10.1186/s13065-023-01087-x

**Published:** 2023-11-24

**Authors:** Khalid A. M. Attia, Ebrahim A. El-Desouky, Amr M. Abdelfatah, Nahla A. Abdelshafi

**Affiliations:** 1 Pharmaceutical Analytical Chemistry Department, Faculty of Pharmacy, Al-Azhar University, Nasr City, Cairo, 11751 Egypt; 2 Department of Pharmaceutical Analytical Chemistry, School of Pharmacy, Badr University in Cairo, Badr City, Cairo, 11829 Egypt

**Keywords:** Levamisole, Triclabendazole, Thin layer chromatography (TLC)–densitometry, High performance liquid chromatography (HPLC), Anthelmintic drugs, Green assessment

## Abstract

Two simple and rapid chromatographic methods were developed and validated for the analysis of levamisole and triclabendazole simultaneously in pure and pharmaceutical products. The first method is thin-layer chromatography (TLC) with densitometry, and the second method is high-performance liquid chromatography with PDA detection (HPLC–PDA). A Hypersil BDS C18 column with dimensions of 4.6 × 150 mm and a particle size of 5 µm was used in the HPLC–PDA method. An isocratic condition was used to carry out the separation, and the mobile phase was made up of acetonitrile and a 0.03 M potassium dihydrogen phosphate buffer in double-distilled water. The ratio of the mobile phase preparation was 70:30 (v/v), and the flow rate was 1 mL/min. A wavelength of 215 nm was employed for analyte detection. Precoated silica gel 60 F254 aluminium plates were used for the TLC method's separation. Mobile phase was made of ethyl acetate, hexane, methanol, and ammonia (69:15:15:1) for the separation. The detection wavelength selected was 215 nm. According to the International Council for Harmonization (ICH) guidelines, the proposed methods were validated and it was found that the two chromatographic methods are accurate, precise, and linear for both compounds in the range of 3.75–37.5 and 6–60 mg/L for the HPLC method for levamisole and triclabendazole, respectively and in the range of 2–14 µg/spot for the TLC method. The developed methods greenness profile was assessed using AGREE and ComplexGAPI tools.

**Graphical Abstract:**

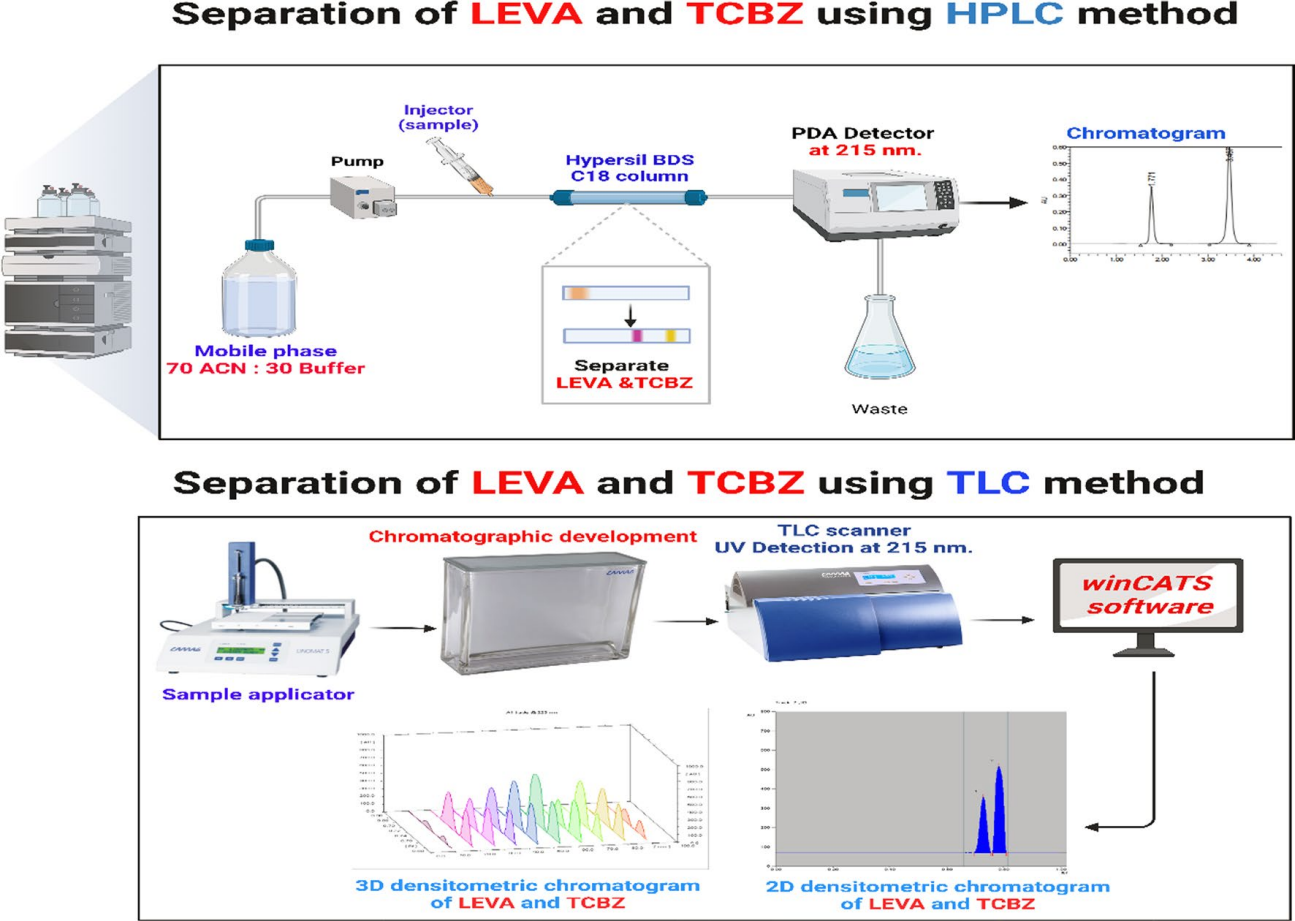

## Highlights

1. Development of two chromatographic methods for simultaneous analysis of binary anthelmintic mixture.

2. HPLC-PDA using isocratic conditions and TLC-Densitometry techniques used for the separation of levamisole (LEVA) and triclabendazole (TCBZ).

3. The developed methods were validated according to ICH guidelines and green assessment using AGREE and ComplexGAPI tools.

## Introduction

**Fig. 1:**
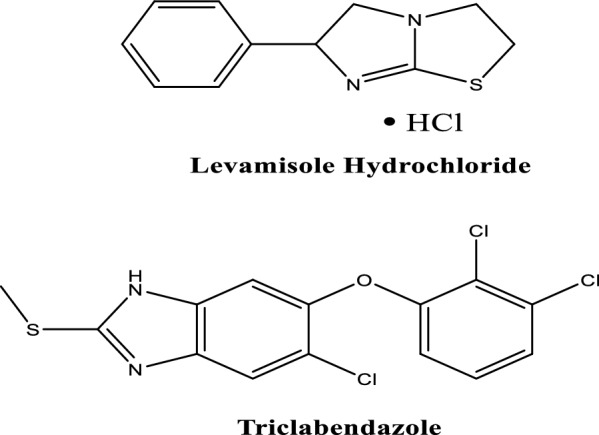
Levamisole hydrochloride and Triclabendazole chemical structures

One of the primary factors causing economic losses in the sheep breed business is intestinal nematode infections [1, 2]. Veterinarians are now employing a variety of anthelmintic medication in combination to promptly treat animals as the incidence of parasite resistance continues to rise [2]. Martibendazene is an oral suspension formula that consists of two active ingredients with distinct pharmacological actions on sheep GIT worms [3, 4]. The anthelmintic drugs used are levamisole hydrochloride (LEVA) and triclabendazole (TCBZ), their chemical structures are illustrated in Fig. 1. The concurrent administration of LEVA with TCBZ has been observed to result in superior therapeutic outcomes and accelerated amelioration of hepatic pathologies in naturally infected sheep afflicted with Fasciola species [4].

LEVA (C_11_H_12_N_2_S) is effectively eradicating the parasitic infection known as ascariasis in the human population. Additionally, it exhibits activity against the hookworm parasite, whereas LEVA demonstrates limited efficacy in treating enterobiasis and trichuriasis. The racemic form tetramisole is less effective at killing worms than LEVA [5]. In 1990’s, the FDA approved the use of Levamisole as a colon cancer adjuvant therapy [6]. Previously, levamisole was employed as a therapeutic agent for rheumatoid arthritis [7]. The findings support claims that levamisole has immunomodulatory qualities that make it effective for improving immunological response even in severely compromised folks [8, 9]. The trial also showed that LEVA is clinically effective in treating people with mild coronavirus infection (COVID-19) [10].

TCBZ (C_14_H_9_C_l3_N_2_OS), is a type of benzimidazole anthelmintic that is specifically recommended for the treatment of sheep and other types of cattle. In ovine and bovine animals, this treatment is effective in eliminating early immature and mature Fasciola species [11, 12]. Triclabendazole's effectiveness against Fasciola infections in livestock has been documented since the 1980’s [13]. Following oral administration, the triclabendazole that is absorbed cannot be identified in the plasma due to its rapid clearance by the liver. The liver metabolizes the triclabendazole into triclabendazole sulfoxide and triclabendazole sulphone [14]. Since the dosage form of LEVA with TCBZ is new and to simultaneously analyze both medications in the new dosage form, we had to design new methodology and validate it.

For the quantitative determination of LEVA by itself or with the combination of other medications either in its pure form or in dosage forms or its degradation form or in the presence of metabolites, a variety of techniques have been documented. These techniques include high-performance liquid chromatography coupled with ultraviolet detection (HPLC–UV) [14–26], with mass spectrometry detection (HPLC–MS/MS) [27–29], liquid chromatography coupled with ultraviolet detection (LC-UV) [30], with mass spectrometry detection (LC–MS/MS) [31–34], ultra-performance liquid chromatography (UPLC) [35], gas chromatography/mass spectrometry (GC–MS) [36–38], high performance thin-layer chromatographic methods (TLC) [38, 39], capillary electrophoresis [38, 40, 41], spectrophotometric methods [42], potentiometric methods [43, 44], electrochemiluminescence [45], electro-membrane extraction [46], and electrochemically using electrodes modified with boron-doped diamond [47]. The concentration of TCBZ alone or in combination with other medications has been measured using a variety of techniques, including high performance liquid chromatography coupled with ultraviolet detection (HPLC–UV), pharmaceutical dosage forms, biological fluids, and in the presence of its metabolites [48–54], with fluorescence detection [55], liquid chromatography coupled with fluorescence detection [56], with mass spectrometry detection (LC–MS/MS) [57, 58], spectrofluorometric method [59], and spectrophotometric methods [60]. To our knowledge, none of these techniques quantitively analyzed both drugs except one spectrophotometric method [61].

In analytical chemistry laboratories, the optimization of chromatographic conditions to quantitatively analyse the binary mixture is a challenging undertaking, this requires numerous trials conducted by experienced analysts, as well as scientific predictions of how the drugs will behave chromatographically corresponding to their structures. The main aim of this study is the development of robust, sensitive, and optimized chromatographic techniques for determining LEVA and TCBZ simultaneously either in pure forms or dosage form. The present study aims to develop with optimized conditions of reversed-phase (RP)-HPLC coupled with photodiode array detector (PDA) and TLC-densitometry methods. These methods aim to facilitate the rapid separation of the investigated compounds. The proposed methods were fully validated and statistically analyzed according to ICH parameters [62]. For quality control analysis of the aforementioned pharmaceuticals’ with the lack of interference of excipients, it can be applied for regular analysis in a variety of pharmaceutical companies.

Calculating the greenness profile of two the methods were performed using AGREE and ComplexGAPI softwares. AGREE which is abbreviation of Analytical GREEnness metric approach consists of 12 principles in green analytical chemistry, where the weight of each principle can be adjusted and varied for confident flexibility. The 12 principles are presented in a clockwise diagram where each sector is colored scaled from red to yellow to green representing the greenness of each principle in the method. The greener the diagram, the score tends to be near one [63–65].

An advanced tool for assessing how analytical processes affect the environment is the ComplexGAPI green assessment tool. It encompasses every facet of the process, including the synthesis and production of materials needed for the technique as well as sample collection, preparation, and analysis. The tool represents every phase of the process and how they impact on the environment employing a pictogram that consists of a hexagon and five pentagrams. To indicate a low, medium, or high effect, the pentagrams are colored green, yellow, or red, respectively. If specific conditions are satisfied, such having renewable or biodegradable materials used in the process, the hexagon is colored green. The tool is used for enhancing analytical chemistry's sustainability which can be used to track improvements over time and pinpoint places where more environmentally friendly analytical processes can be implemented [66, 67].

Our aim to develop a new method to quantitively separate LEVA and TCBZ using RP-HPLC and TLC-Densitometry. The developed method aims to fulfil the ICH parameters requirements and to be green to maintain sustainability.

### Materials and chemicals

LEVA and TCBZ working standards were generously given by (Pharma Swede Ph. Co., 10th of Ramadan City, Egypt). The reported purities of the LEVA and TCBZ were 99.7% and 99.6%, respectively. Martibendazene^®^ oral suspension manufactured by Martiros for pharmaceutical industrial Co., labeled to contain 7.5 gm LEVA and 12 gm TCBZ for each 1 mL, was provided by Martiros for pharmaceutical industrial Co. Throughout the entire study, HPLC grade solvents and reagents were used. We obtained acetonitrile (ACN) and methanol of HPLC grade obtained from (Sigma-Aldrich, Germany), orthophosphoric acid from (Merck, Germany), and potassium dihydrogen phosphate (KH_2_PO_4_) from (LOBA Chemie, India), ethyl acetate and hexane from (PIOCHEM Co., Egypt), and all provided of analytical grades. Hydrochloric acid and sodium hydroxide (El-Nasr Company, Egypt) prepared as 0.5 M aqueous solutions. Hydrogen peroxide 30% (TopChem Company, Egypt) Double distilled water (Otsuka Pharmaceutical Co., Cairo, Egypt).

## Experimental

### Materials and chemicals

LEVA and TCBZ working standards were generously given by (Pharma Swede Ph. Co., 10th of Ramadan City, Egypt). The reported purities of the LEVA and TCBZ were 99.7% and 99.6%, respectively. Martibendazene^®^ oral suspension manufactured by Martiros for pharmaceutical industrial Co., labeled to contain 7.5 gm LEVA and 12 gm TCBZ for each 1 mL, was provided by Martiros for pharmaceutical industrial Co. Throughout the entire study, HPLC grade solvents and reagents were used. We obtained acetonitrile (ACN) and methanol of HPLC grade obtained from (Sigma-Aldrich, Germany), orthophosphoric acid from (Merck, Germany), and potassium dihydrogen phosphate (KH_2_PO_4_) from (LOBA Chemie, India), ethyl acetate and hexane from (PIOCHEM Co., Egypt), and all provided of analytical grades. Hydrochloric acid and sodium hydroxide (El-Nasr Company, Egypt) prepared as 0.5 M aqueous solutions. Hydrogen peroxide 30% (TopChem Company, Egypt) Double distilled water (Otsuka Pharmaceutical Co., Cairo, Egypt).

### Instrumentation and chromatographic conditions

Waters Alliance 2690 HPLC module conducted with a column compartment, auto sampler, degasser, and quaternary pump, coupled with Waters 996 Photodiode Array Detector (PDA) was used. Empower3^®^ chromatographic software (Empower 3 Software Build 3471 SPs) was used to process the obtained results. pH meter (Jenway 3510, UK), electronic balance (Vibra, Japan), and membrane filter (0.45 µm, Millipore, Ireland) were used. The analytical column utilized in the experiment was a Hypersil BDS C18 column (4.6 × 150 mm, 5 µm). Both the calibration of the data and the computation of the regression equation were done using Microsoft Excel 365. The structures of the analytes were sketched using ChemBioDraw Ultra 14.0 software. Under isocratic condition, the analysis was carried out using mobile phase system made of ACN and 0.03 M KH_2_PO_4_ (70:30 v/v) in double distilled water, orthophosphoric acid was used to keep pH (3). Mobile phase solvents were pumped at a flow rate of 1 mL/min after being filtered then sonicated for degassing for 15 min before to use. The run time was 4.5 min with ambient column temperature. The equilibration of the analytical column lasted for 30 min. using the mobile phase followed by injection of the prepared sample (10 µL). Wavelength maximum was observed at 215 nm.

The HPTLC densitometer device consisted of Camag^®^ Linomat five autosampler with Camag^®^ micro syringe 100 µL (Muttenz, Switzerland). The stationary phase was made of (20 × 10 cm^2^) aluminium sheets and coated with (60 F254) silica gel (Merck, Darmstadt, Germany), where separation was accomplished. Densitometric scanning was performed using A CAMAG scanner (3S/N 1302139; Muttenz, Switzerland) with win CATS® software version 1.4.2.8121. The application of samples onto the thin-layer chromatography (TLC) plates was performed in a quantitative manner using the Camag^®^ Linomat autosampler, employing a 100µL micro syringe. The bands, with a length of 6 mm, were spotted with 10.5 mm distance from each spot and 15 mm from the bottom border of the plate. The optimal components of the mobile phase utilized for the chromatographic separation was ethyl acetate: hexane: methanol: ammonia (69:15:15:1, by volume). The plates were developed in an ascending chromatographic chamber and the pre-saturation lasted for 60 min. with the mobile phase at 25 ℃, eight cm from the spotting line. The generated bands were scanned with a UV lamp adjusted at 215 nm at 20 mm/s for densitometric analysis after the plates were left at room temperature for 30 min. to dry.

### Preparation of standard and working solutions

Concentration of stock solution of (750 mg/L) for LEVA and (1200 mg/L) for TCBZ were prepared by dissolving of 75 mg of LEVA and 120 mg of TCBZ in methanol up to 100-mL volumetric for HPLC. Followed by aliquoting 10 mL of the stock solution into 100-mL volumetric flask and diluted with the mobile phase While concentration of stock solutions (1000 mg/L) for each drug was determined by accurately weighing and transferring aliquots of 10 mg each of LEVA and TCBZ into 10-mL volumetric flasks using methanol for TLC–Densitometry. The volume of the all the solutions was brought up to the mark with the same diluting solvent.

### Validation procedure

In the development and validation of the chromatographic procedures, linearity, range, accuracy, precision, limit of detection (LOD), limit of quantification (LOQ), robustness, and system suitability were produced [62].

#### Linearity

For HPLC, the calibration curve was plotted using concentrations ranging from 3.75 to 37.5 mg/L for LEVA and 6 to 60 mg/L for TCBZ against the observed peak area. Measurements were performed in triplicates and linearity was established by applying the linear regression analysis. While TLC method was performed by accurately applying volumes of each drug stock solution onto TLC sheets. This resulted in spot volumes of 2–14 µg/spot for both LEVA and TCBZ. Linear regression equations were obtained by constructing the calibration curve as the obtained peak area of each drug against their concentrations.

#### Accuracy

The accuracy of the measurements was calculated using nine determinations over three different concentrations of LEVA and TCBZ apart from the linearity ranges. The percent recovery for the prepared concentration was used to calculate accuracy, which was then represented as the percent recoveries mean ± standard deviation (SD). The tested concentrations in (HPLC) method were 15, 22.5, and 30 mg/L for LEVA, and 12, 24, and 48 mg/L for TCBZ. The aforementioned procedures were performed to determine various concentrations, with each measurement being observed and linear regression equation was applied. In the thin-layer chromatography (TLC) method, the concentrations of LEVA and TCBZ were observed to be 6, 8, and 12 µg/spot and 4, 6, and 10 µg/spot, respectively.

#### Precision

Assessment of the precision was by performing triplicate measurements over a period of three consecutive days using three distinct concentration levels within range obtained of each standard (the inter-day precision). While intra-day precision is the ability to repeat the measurements of three concentrations on the same day. The three evaluated concentrations using the HPLC technique were 15, 22.5, and 30 mg/L for LEVA and 12, 24, and 48 mg/L for TCBZ. The three evaluated concentrations using the TLC technique were 6, 8, and 12 µg/spot for LEVA and 4, 6, and 10 µg/spot for TCBZ. The chromatographic procedures mentioned above were used to determine different concentrations.

#### Detection and quantitation limits

The limit of detection (LOD) and limit of quantification (LOQ) for both methods were determined for LEVA and TCBZ. The detection limit was computed as (3.3* SD of intercept)/slope [62] while quantification limit as (10* SD of intercept)/slope [62].

#### Robustness and system suitability analysis

To determine whether the developed methods were robust or not, the relative standard deviation (RSD) was evaluated by performing the HPLC method with altering mobile phase ratio and flow rate with minute changes. While for TLC method, the variation was the introduction of minor modifications to the mobile phase ratio and detector wavelength. The parameters for system suitability testing were evaluated with respect to the selectivity and tailing factor, theoretical plate number, and resolution.

#### Forced degradation study

All degradation experiments were performed with drug solutions of (100 mg/L) concentration for each drug. Triclabendazole and levamisole were stressed under the following conditions: 0.5 M HCl, 0.5 M NaOH, 30% H_2_O_2_, at 100 °C. The stressed samples were injected into the HPLC. Standards of triclabendazole and levamisole were reacted with 0.5 M HCl for 2 h and 0.5 M NaOH for 5 h. They were also treated with 30% H_2_O_2_ for 30 min. and heating at 100 °C for 1h.

1. Heating condition, transfer 5 mL of solutions concentration of (100 mg/L) to a 50-mL volumetric flask, and the required volume was completed with methanol. The solutions of TCBZ and LEVA were refluxed at 100 °C for 1 h. The solutions were injected into HPLC after cooling down.
2. Alkaline, acidic, and oxidative conditions, transfer 5 mL of solutions concentration of (100 mg/L) to a 50-mL volumetric flask with adding 5 mL of 0.5 M NaOH to TCBZ and LEVA. Then, the solutions were refluxed at 100 °C for 5 h. After cooling the solutions were neutralized with 0.5 M HCl. The above procedure was replicated by 0.5 M HCL instead of NaOH while heating for 2 h and finally neutralized using 0.5 M NaOH. The oxidative conditions applied to TCBZ and LEVA was evaluated by repeating the above procedure using 5 ml of 30% H_2_O_2_ while refluxing at 100 °C for 30 min. The degraded formed solutions were injected into HPLC for analysis.

### Application to pharmaceutical dosage form

Martibendazene^®^ oral suspension as directed in the labelling each 1 ml contains 7.5 gm of levamisole and 12 gm of triclabendazole. A dilution of 1:100 was prepared using methanol, the suspension was sonicated for 15 min till complete dissolving then filtered, the solution was completed to 100 mL.

- HPLC method: The prepared solution was diluted with mobile phase by transfer 1.0 mL of the prepared solution to a 100-mL volumetric flask to obtain (75 mg/L) of LEVA and (120 mg/L) of TCBZ. Under the optimized chromatographic conditions, separation was obtained.
- The solution was spotted onto the TLC plate using the TLC densitometry method. The chromatographic conditions stated above were used for the evaluation and measurement.

The standard addition method was used for both techniques. The evaluation was done using Martibendazene^®^ oral suspension and known exact amounts of each standard were spiked to the dosage form. The recovery evaluation was conducted for each standard using three different addition concentration amounts.

## Results and discussion

### Development and optimization of conditions

For the simultaneous determination of LEVA and TCBZ, two chromatographic techniques are proposed to be developed and validated in the current study.

**Fig. 2:**
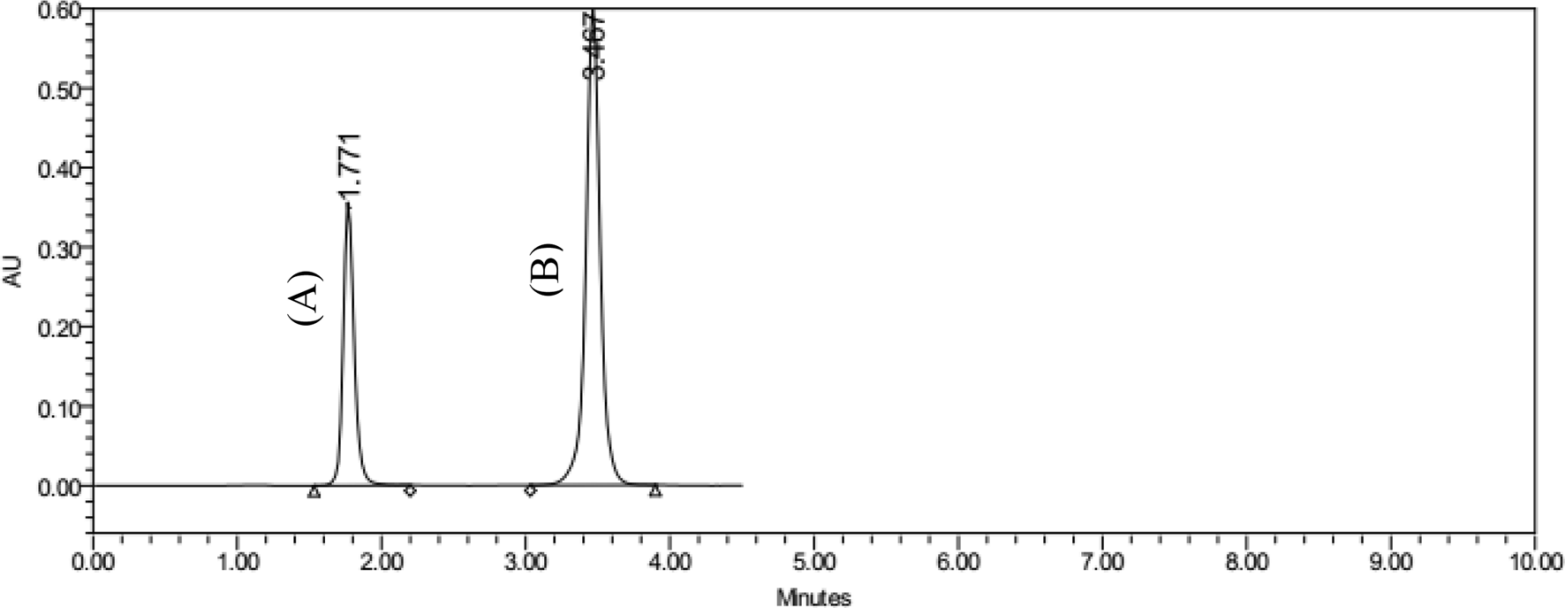
HPLC chromatograms of (**A**) LEVA (37.5 mg/L) and (**B**) TCBZ (60 mg/L) under optimized conditions

**Fig. 3:**
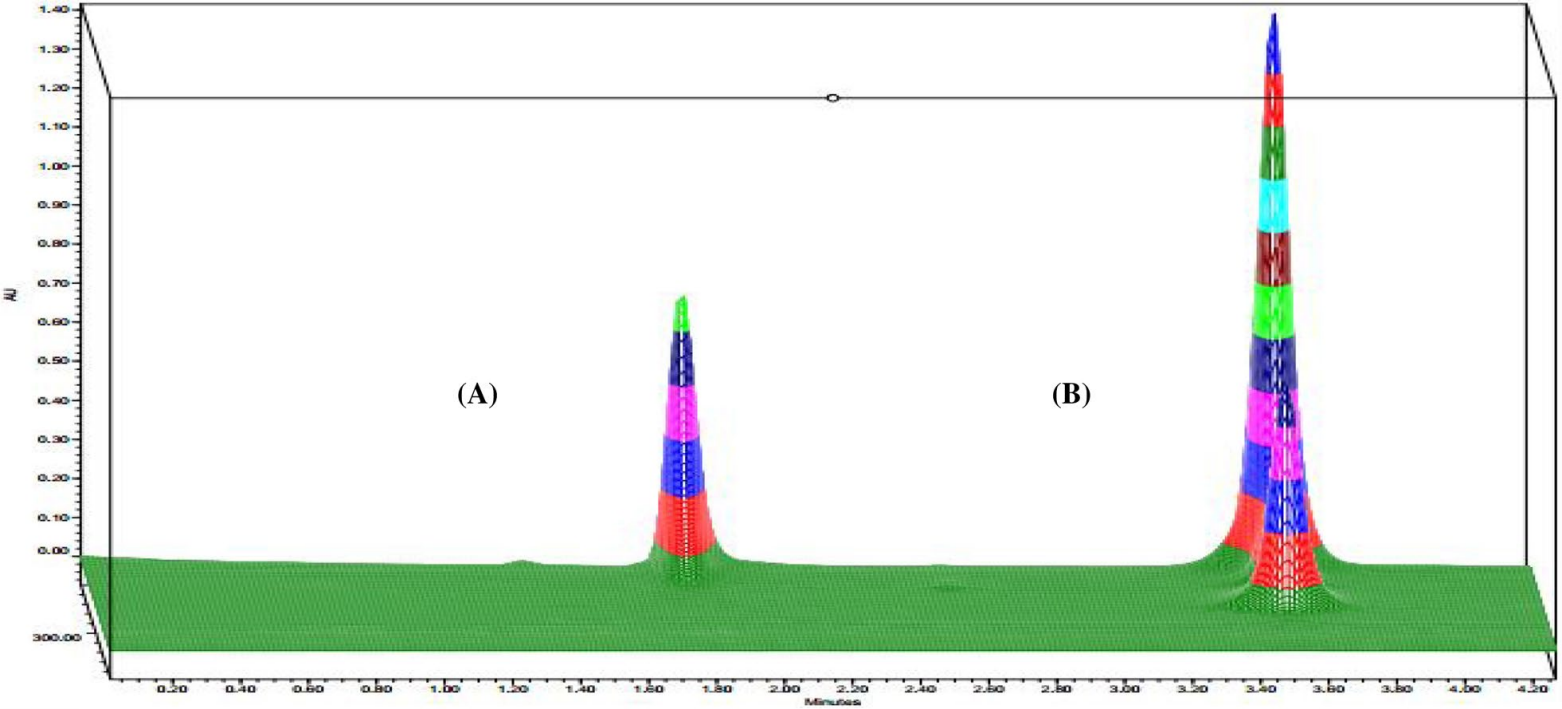
Three-dimension HPLC chromatograms of (**A**) LEVA (37.5 mg/L) and (**B**) TCBZ (60 mg/L) under optimized conditions

Many different mobile phase combinations were tried during HPLC technique development, including water/MeOH, water/ACN, and acetic acid/MeOH, but all of them resulted in either a loss of resolution or an excessively lengthy analysis time. ACN were mixed with KH_2_PO_4_ buffer as a mobile phase in different ratios such as (60:40, 70:30, 80:20). The ratio (70:30) of ACN: KH_2_PO_4_ resulted in a better resolution. The flow rate was alternated to avoid tailing and fronting of the peak, 0.5, 0.8, 1, 1.2, 1.5 mL/min were applied, and the optimized peaks were resulted from 1 mL/min. flow rate. For the selection of wavelength, wavelength maximum (λ_max_) of the spectra of both drugs were selected to carry on further measurement. The optimized chromatographic parameters were obtained by utilizing a combination of ACN and a 0.03 M KH_2_PO_4_ buffer in (70:30) ratio with flow rate (1 mL/min). The optimal separation was attained as shown in Figs. 2–3.

**Fig. 4:**
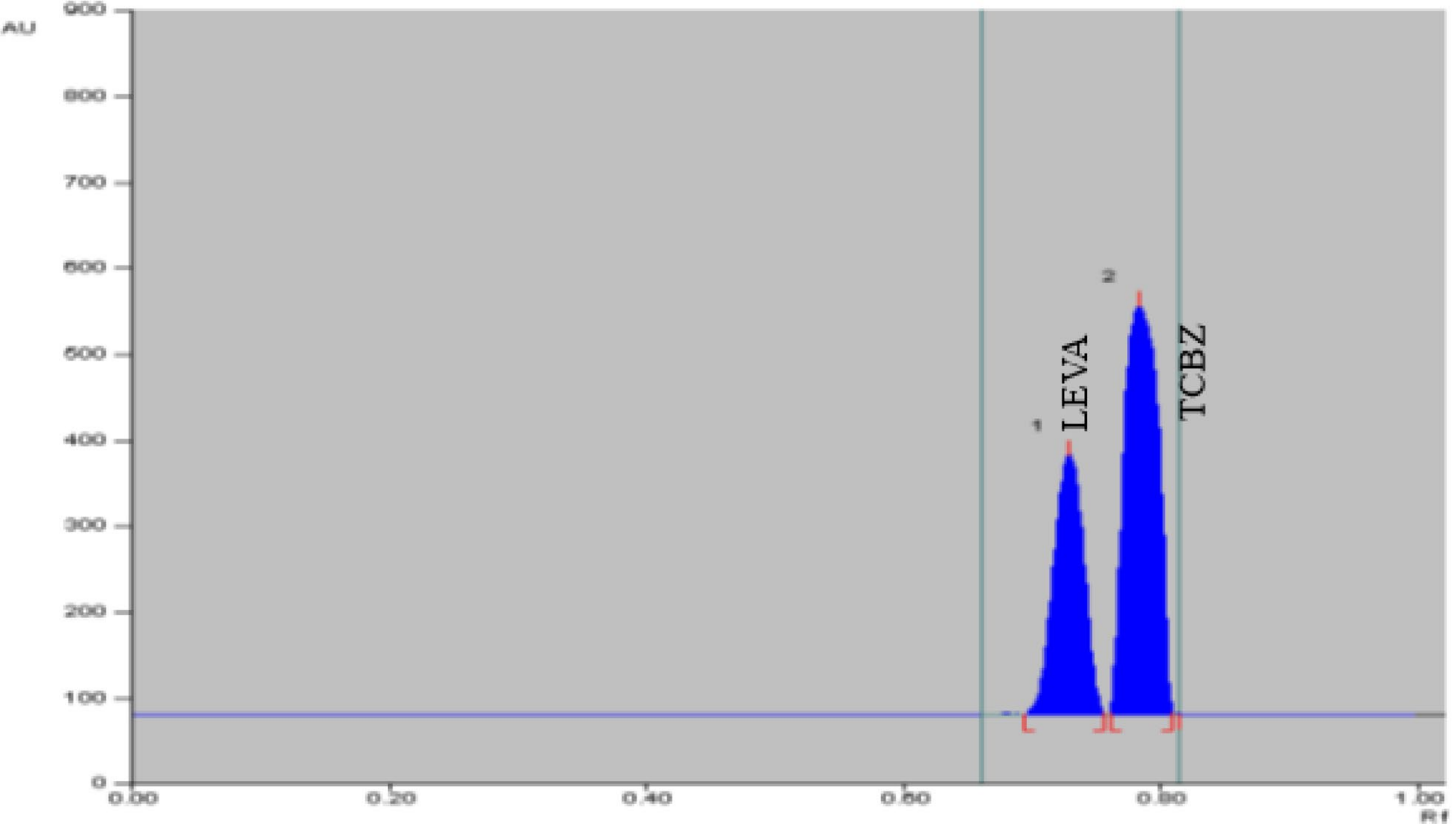
Two dimensions TLC chromatogram of LEVA (7.5 µg/spot) and TCBZ (12 µg/spot) under optimized conditions for chromatography

In the TLC method, the optimization of mobile phase was performed by applying different ratios of methanol: dichloromethane (DCM); (90:10 and 80:20). The obtained spots were overlapped ensuring no separation using this system. Another system was tried using ethyl acetate and hexane, ratios of (80:20 & 95:5 of ethyl acetate: methanol). Spots started to be separated, therefore this system was chosen for the optimization of mobile phase. Hexane and ammonia were added to enhance separation of spots by alternating the polarity of the system. Ratios applied of ethyl acetate, hexane, methanol, and ammonia were (79:10:10:1, 69:15:15:1, and 79:5:15:1). For wavelength selection different wavelength were applied such as 215, 223, 245, 254, and 270 nm. The best chromatographic separation was done with ethyl acetate, hexane, methanol, and ammonia in the proportions (69:15:15:1, by volume) at 215 nm. Demonstrating successful chromatographic separation of LEVA and TCBZ as presented in Fig. 4.

### Method validation

Evaluations of the validation parameters for the two methodologies were presented and evaluated in fulfilment with ICH recommendations [62], as shown in Table 1.

**Table 1:** Validation parameters of the recommended chromatographic methods for determination of LEVA and TCBZ

Items	HPLC		TLC	
	LEVA	TCBZ	LEVA	TCBZ
Wavelength (nm)	215 nm		215 nm	
Linearity range (mg/L)	3.75–37.5 mg/L	6–60 mg/L	2–14 mg/L	2–14 mg/L
Intercept	32507	35799	1175.3	3700.1
Slope	49529	69627	1201.5	922.73
Correlation coefficient (r^2^)	0.9999	0.9999	0.9998	0.9998
Accuracy (Mean ± SD)	99.78 ± 1.8	99.70 ± 1.0	100.11 ± 0.95	100.12 ± 0.67
Precision				
Repeatability (% RSD)	0.58	0.62	1.1	0.56
Intermediate precision **(**% RSD)	0.64	0.67	1.1	0.58
LOD (mg/L)	0.712	1.099	0.538	0.446
LOQ (mg/L)	2.157	3.331	1.632	1.353

#### Linearity

In the HPLC method, LEVA and TCBZ showed linearity in the ranges of 3.75–37.5 and 6–60 mg/L, respectively, whereas in the TLC method, LEVA and TCBZ showed range linearity of 2–14 µg/spot for the binary mixture. The (r^2^) was greater than 0.9998 that is more than 0.995 required for linearity [68]. The obtained calibration curves were used to construct linear regression equations.

#### Accuracy

The accuracy of the proposed methods was evaluated by calculating the mean percentage recovery and standard deviation (SD). In the HPLC method the mean values, along with their corresponding standard deviations, were determined to be 99.78 ± 1.8 and 99.70 ± 1.02 for LEVA and TCBZ, respectively. In the TLC method, the observed values for LEVA were, 100.11 ± 0.95 while for TCBZ they were 100.12 ± 0.67. Both methods were determined to be accurate since the results were in the range of 98–102% as shown in Table 1.

#### Precision

Upon evaluating repeatability, % RSD was found to 0.58 and 0.62 for LEVA and TCBZ, respectively using the HPLC method and 1.1 for LEVA and 0.56 for TCBZ using the TLC method. In order to assess the precision of measurements over multiple days, inter-day precision testing was conducted, % RSD was found to be 0.64 and 0.67 for LEVA and TCBZ, respectively, using the HPLC method and 1.05 for LEVA and 0.58 for TCBZ using the TLC method. The data presented in this study indicate that both methods exhibit a high level of precision. The results expressed as relative standard deviations (% RSD) as shown in Table 1 indicating the precision of the methods since % RSD doesn’t exceed 2%.

#### LOD and LOQ

By computing the (LOD) and (LOQ) equations previously stated, the corresponding results are shown in Table 1, the obtained results showed sensitivity.

#### Robustness and system suitability parameters

The assessment of robustness involved the evaluation of the effects of making small modifications to the chromatographic conditions of the mobile phase ratio (ACN, 70 ± 1 v/v) and flow rate (1 ± 0.1 mL/min) in the HPLC method. In the TLC method, minute changes in the ratio of the mobile phase ratios (ethyl acetate, 69 ± 1%) and detector wavelength (215 ± 3 nm). The two methods exhibited robustness as the examined variables did not show significant change in the results proven by the calculate relative standard deviation (% RSD) as shown in Tables 2 and 3. For the chromatographic procedures, system suitability metrics such as peak resolution, capacity, selectivity, and tailing factor were computed as shown in Tables 4 and 5. A comparison between the proposed method with previously reported methods were presented in Table 6.

**Table 2:** Parameters involved in evaluating the robustness of the developed HPLC method

Drug	Parameters		Retention time (t_R_)	Capacity factor (K´)	Resolution (R)	Tailing factor (T)
Levamisole	Flow rate	1.1 mL/min	1.757	0.76	–-	1.19
		1 mL/min	1.771	0.77	–-	1.17
		0.9 mL/min	1.823	0.82	–-	1.19
	Mobile phase ratio (ACN: KH_2_PO_4_)	69:31	1.796	0.80	–-	1.19
		70:30	1.771	0.77	–-	1.17
		71:29	1.804	0.80	–-	1.19
Triclabendazole	Flow rate	1.1 mL/min	3.355	2.36	10.75	0.99
		1 mL/min	3.467	2.47	11.40	0.99
		0.9 mL/min	3.499	2.50	11.17	0.98
	Mobile phase ratio (ACN: KH_2_PO_4_)	69:31	3.505	2.50	11.42	0.97
		70:30	3.467	2.47	11.40	0.99
		71:29	3.314	2.31	10.27	0.98

**Table 3:** Parameters involved in evaluating the robustness of the developed TLC method

Drug	Parameters		Retention time (t_R_)	Capacity factor (K´)	Resolution (R)	Tailing factor (T)
Levamisole	Detector wavelength	212 nm	0.72	0.395	–-	0.8
		215 nm	0.71	0.408	–-	0.8
		218 nm	0.71	0.408	–-	0.8
	Mobile phase ratio ethyl acetate: hexane: methanol: ammonia	68:15:16:1	0.71	0.395	–-	0.8
		69:15:15:1	0.71	0.408	–-	0.8
		70:15:14:1	0.71	0.408	–-	0.8
Triclabendazole	Detector wavelength	212 nm	0.76	0.301	1.29	0.9
		215 nm	0.77	0.298	1.33	1
		218 nm	0.78	0.296	1.31	0.9
	Mobile phase ratio ethyl acetate: hexane: methanol: ammonia	68:15:16:1	0.74	0.296	1.28	0.9
		69:15:15:1	0.77	0.298	1.33	1
		70:15:14:1	0.76	0.301	1.32	1

**Table 4:** System suitability parameters for the developed HPLC method

Parameters	Obtained value		Reference value[69]
	Levamisole	Triclabendazole	
Resolution	11.40		> 2
α "relative retention"	3.20		> 1
K` "capacity factor"	0.77	2.47	K` > 2
N "column efficiency"	2739.14	2739.14	> 2000
Tailing factor	1.17	0.99	= 1 for the ideal peak

**Table 5:** System suitability parameters for the developed TLC method

Parameters	Obtained value		Reference value[70]
	Levamisole	Triclabendazole	
Resolution	1.33		> 1
α "relative retention"	1.37		> 1
K` "capacity factor"	0.408	0.298	K` > 1
Tailing factor	0.8	1	= 1 for the ideal peak

**Table 6:** Comparative study of the proposed method with the previously reported methods

Parameters	Reported Method 1 [71]		Reported Method 2 [72]	Reported Method 3 [73]		Proposed Method			
Techniques	Spectrophotometry		LC–MS/MS	Chemometric		HPLC		TLC	
Drugs	TCBZ	LEVA	Mix of ten drugs	TCBZ	LEVA	TCBZ	LEVA	TCBZ	LEVA
Linearity	1–10 Or 2–20 mg/L	2–14 mg/L	0–500 µg/L (linearity rang for mix of ten drugs)	1–9 mg/L	5–25 mg/L	6–60 mg/L	3.75 -37.5 mg/L	2–14 µg/spot	2–14 µg/spot
LOD	0.08 mg/L	0.19 mg/L	Less than 1 µg/L	––-	––-	1.09 mg/L	0.71 mg/L	0.44 mg/L	0.53 mg/L
LOQ	0.23 mg/L	0.58 mg/L	–––	––-	––-	3.33 mg/L	2.15 mg/L	1.35 mg/L	1.63 mg/L
Application	Veterinary formulation		Milk	Veterinary formulation		Pharmaceutical dosage form			

#### Specificity of the proposed method

**Fig. 5:**
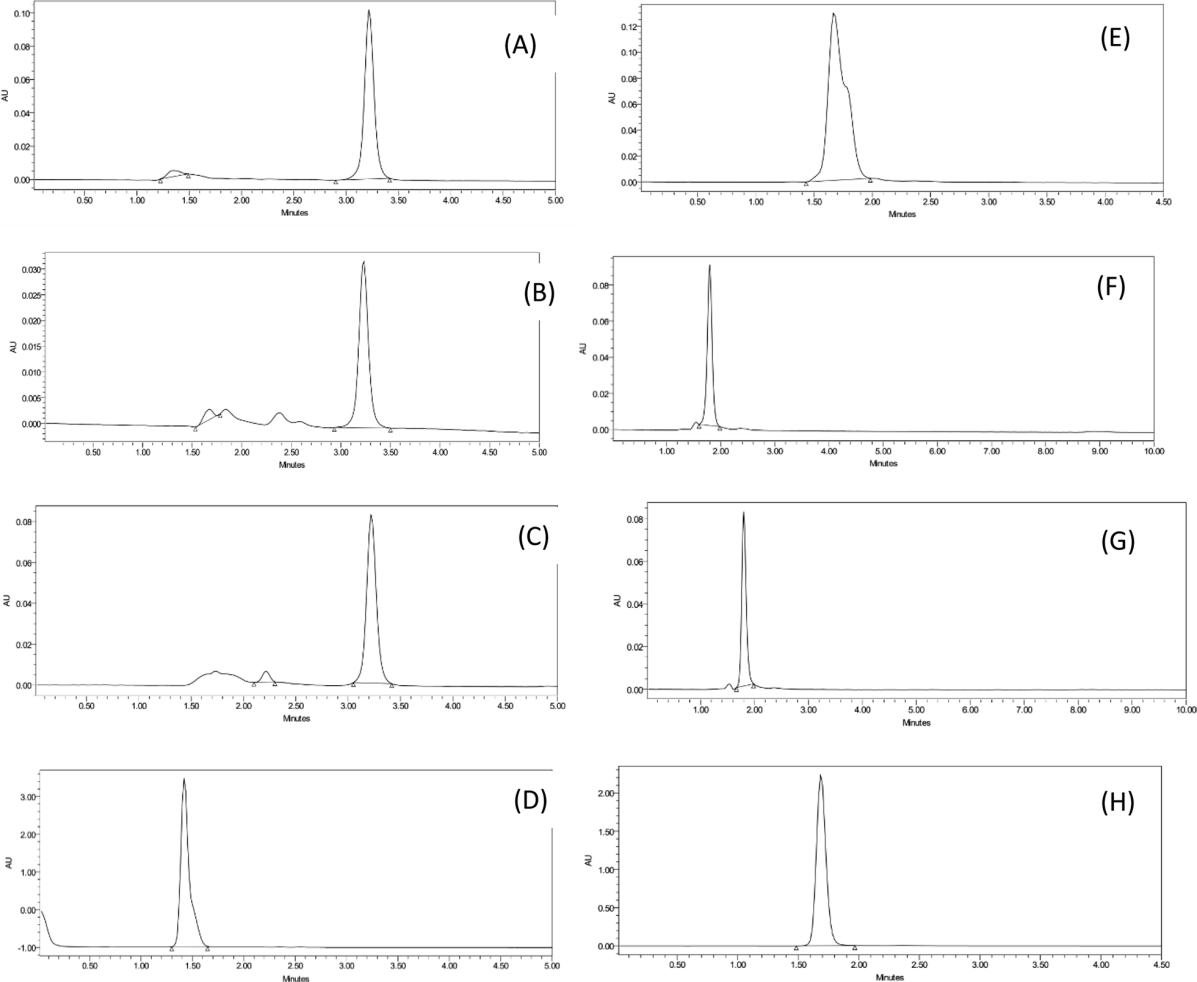
Chromatograms of forced degradation and stability studies: **A** TCBZ degraded with 0.5 M NaOH; **B** TCBZ degraded with 0.5 M HCl; **C** TCBZ degraded with heating; **D** TCBZ degraded with 30% H_2_O_2_ for 30 min; **E** LEVA degraded with 0.5 M NaOH; **F** LEVA degraded with 0.5 M HCl; **G** LEVA degraded with heating; **H** LEVA degraded with 30% H_2_O_2_

By applying forced degradation to both drugs, TCBZ was completely degraded using H_2_O_2_ but partially degraded with HCl, NaOH, and temperature. The obtained chromatograms showed the specificity of the method to separate between the drug and its degraded form. The retention times (t_R_) of the degradation forms differs from LEVA t_R_. However, by following the same degradation procedures for LEVA, the drug was not degraded. Therefore, when a mixture of TCBZ and LEVA was exposed to the previous stress conditions, the proposed method will successfully separate each peak with quantitative measurements. Chromatograms of each drug with each degradation are presented in Fig. 5.

### Assay of pharmaceutical dosage form

For the analysis of LEVA and TCBZ in Martibendazene+ oral suspension, both methods were applied. The developed procedures for determining LEVA and TCBZ selectively in the presence of excipients were validated using the standard addition technique, and the findings were acceptable. Mean of % R ± SD of 99.59 ± 0.47 and 100.42 ± 1.3 for LEVA and TCBZ, respectively (Tables 7 and 8).

**Table 7:** Assessment of LEVA and TCBZ in Martibendazene® oral suspension by the developed HPLC method and outcomes of standard addition method

Product	Drugs	Standard addition				
Martibendazene® Suspension^**a**^		Claimed taken	Added	Total Found^b^	Standard found^b^	Recovery %^b^
	Levamisole	7.5	––	7.48	–-	99.75
		7.5	3.75	11.21	3.73	99.38
		7.5	7.50	14.92	7.44	99.25
		7.5	15	22.50	15.02	100.12
			**Mean ± SD**			**99.59 ± 0.47**
	Triclabendazole	12	–-	11.95	–-	99.59
		12	6	17.91	5.96	99.33
		12	12	24.16	12.21	101.78
		12	24	35.99	24.04	100.15
			**Mean ± SD**			**100.42 ± 1.3**

^a^ Labeled to have 75 mg LEVA and 120 mg TCBZ

^b^ Average of triplicate measurements

The bold values express (Mean) which is the average of recovery percentage and (SD) which is the standard deviation of recovery percentage.

**Table 8:** Assessment of LEVA and TCBZ in Martibendazene^®^ oral suspension by the developed TLC method and outcomes of standard addition technique

Product	Drugs	Standard addition				
Martibendazene® Suspension^**a**^		Claimed taken	Added	Total Found^b^	Standard found^b^	Recovery %^b^
	Levamisole	4	–-	3.99	–-	99.87
		4	2	5.98	1.99	99.57
		4	4	8.03	4.04	100.99
		4	6	9.98	5.99	99.80
			**Mean ± SD**			**100.12 ± 0.76**
	Triclabendazole	6	–-	6.02	–-	100.32
		6	2	8	1.98	98.79
		6	6	12.01	5.99	99.81
		6	8	14.06	8.04	100.46
			**Mean ± SD**			**99.69 ± 0.85**

^a^ Labeled to have 75 mg LEVA and 120 mg TCBZ

^b^ Average of three measurements

The bold values express (Mean) which is the average of recovery percentage and (SD) which is the standard deviation of recovery percentage.

### Statistical analysis

By comparing t and F values of our developed method and the reported one in the literature, there was no significant difference. Both computed t and F values were with lesser values than the theoretical values. The methods are accurate and precise by applying Student-t-test and F-value at 95% confidence level [74]. This was demonstrated statistically by comparing the results of the HPLC and TLC developed methods to reported ones [60, 75], shown in Table 9.

**Table 9:** shows a statistical comparison of the findings from the examination of Martibendazene® oral suspension using the suggested HPLC and TLC procedures with the methods that have been previously described

Parameters	Levamisole			Triclabendazole		
	Proposed HPLC method	Proposed TLC method	Reported Method [75]	Proposed HPLC method	Proposed TLC method	Reported Method [60]
N*	5	5	5	5	5	5
X̅**	100.74	99.87	99.77	100.46	99.76	100.66
SD	0.924	0.951	0.938	0.961	0.493	0.856
Variance	0.854	0.905	0.879	0.925	0.243	0.733
Student’s-t-test***	1.647 (2.306)	0.175 (2.306)	___	0.360 (2.306)	2.045 (2.306)	___
F-value ***	1.029 (6.388)	1.029 (6.388)	___	1.262 (6.388)	3.016 (6.388)	___

^*^ Number of experiments

^**^ The mean of percent recovery of pharmaceutical preparation

^***^ The values in parenthesis are tabulated values of “t “and “F” at (P = 0.05)

### Assessment of greenness profile of the chromatographic methods

The proposed chromatographic techniques were then evaluated for their greenness using the AGREE (Analytical GREEness) tool [64]. Using a greenness calculator, twelve rules were applied to produce a clock-like graph. AGREE pictogram, showing a score in the centre and assessing the influence on the environment from deep green to deep red. The developed HPLC and TLC-densitometry procedures' AGREE pictograms display scores of (0.71) and (0.8) with flimsy green hues, respectively. The results showed that methods are eco-friendly, and the TLC method is greener than the reported spectrophotometric one (0.71) [61], as shown in Table 10. Through ComplexGAPI (complex green analytical procedure index) [66], it has been shown that the proposed methods have a low risk of environmental damage. A color scale of pictogram with five pentagrams, representing sample preparation, reagent, and solvent use, as well as instrumentation and a hexagonal, representing pre-analysis condition, are used to illustrate the findings. Green color denotes a considerably safer impact on the environment, yellow denotes a problematic impact, and red denotes a risky impact that should be avoided. While the proposed HPLC pictogram produced 10 green colors, three red colors, and two yellow and the proposed TLC pictogram produced 10 green colors, two red colors, and three yellow. The methods are signifying greener more than the reported [61] pictogram produced eight green colors, three red colors, four yellow colors. While the hexagonal HPLC, TLC, reported methods [61] with 1.00 E-factor are all green as shown in Table 10.

**Table 10:** Greenness assessment of developed and reported method using AGREE and ComplexGAPI tools

Tool	HPLC	TLC	Reported method [61]
AGREE [64]			
ComplexGAPI [66]			

## Conclusions

The validated chromatographic methods deliver accurate, precise, repeatable, sensitive, and quantification methods for LEVA and TCBZ based on the aforementioned observations from experiments. The evaluation of LEVA and TCBZ in pure powders and pharmaceutical dosage form was successfully accomplished using the developed TLC and HPLC techniques. The methods were validated according to ICH requirements and showed satisfactory chromatographic characteristics. Both techniques were determined to be suitable for use in quality control laboratories. Although HPLC is the most practical method, TLC-densitometry exhibits a greater sensitivity, is inexpensive, eco-friendly, and allowing the determination of multiple samples quickly.

## Data Availability

Most data generated or analyzed during this study are included in this published.

## References

[1] Rashid MH (2018). Anthelmintic resistance in gastrointestinal nematodes of alpacas (Vicugna pacos) in Australia. Parasit Vectors.

[2] Falzon LC (2014). A systematic review and meta-analysis of factors associated with anthelmintic resistance in sheep. Prev Vet Med.

[3] Le Jambre LF, Martin PJ, Johnston A (2010). Efficacy of combination anthelmintics against multiple resistant strains of sheep nematodes. Animal Production Science.

[4] Tabari MA (2022). Therapeutic efficacy of triclabendazole in comparison to combination of triclabendazole and levamisole in sheep naturally infected with Fasciola sp. J Parasit Dis.

[5] Abongwa M, Martin RJ, Robertson AP (2017). A brief review on the mode of action of antinematodal drugs. Acta Vet.

[6] Mutch RS, Hutson PR (1991). Levamisole in the adjuvant treatment of colon cancer. Clin Pharm.

[7] Runge LA, Pinals RS, Tomar RH (1979). Treatment of rheumatoid arthritis with levamisole: long-term results and immune changes. Ann Rheum Dis.

[8] Abdalla EE (1995). The immunomodulatory effect of levamisole is influenced by postoperative changes and type of lymphocyte stimulant. Cancer Immunol Immunother.

[9] Oladele OA (2012). Effects of levamisole hydrochloride on cellular immune response and flock performance of commercial broilers. Brazilian Journal of Poultry Science.

[10] Roostaei Firozabad A (2021). Efficacy and safety of Levamisole treatment in clinical presentations of non-hospitalized patients with COVID-19: a double-blind, randomized, controlled trial. BMC Infect Dis.

[11] Ashrafi K (2014). Fascioliasis: a worldwide parasitic disease of importance in travel medicine. Travel Med Infectious Dis.

[12] Barrera B (2012). The anthelmintic triclabendazole and its metabolites inhibit the membrane transporter ABCG2/BCRP. Antimicrob Agents Chemother.

[13] Kelley JM (2016). Current threat of triclabendazole resistance in fasciola hepatica. Trends Parasitol.

[14] García JJ (1990). Determination of levamisole by HPLC in plasma samples in the presence of heparin and pentobarbital. J Liq Chromatogr.

[15] Sari P, Razzak M, Tucker IG (2004). Rapid, simultaneous determination of levamisole and abamectin in liquid formulations using HPLC. J Liq Chromatogr Relat Technol.

[16] Wyhowski de Bukanski B, Degroodt JM, Beernaert H (1991). Determination of levamisole and thiabendazole in meat by HPLC and photodiode array detection. Z Lebensm Unters Forsch.

[17] Cholifah S, Farina Kartinasari W, Indrayanto G (2007). Simultaneous HPLC determination of levamisole hydrochloride and anhydrous niclosamide in veterinary powders, and its validation. J Liquid Chromatogr Related Technol.

[18] Sari P (2006). HPLC assay of levamisole and abamectin in sheep plasma for application to pharmacokinetic studies. J Liq Chromatogr Relat Technol.

[19] Marriner S, Galbraith EA, Bogan JA (1980). Determination of the anthelmintic levamisole in plasma and gastro-intestinal fluids by high-performance liquid chromatography. Analyst.

[20] Ali HM (2022). Quantitative analysis of abamectin, albendazole, levamisole HCl and closantel in Q-DRENCH oral suspension using a stability-indicating HPLC-DAD method. Molecules.

[21] Vandamme TF, Demoustier M, Rollmann B (1995). Quantitation of levamisole in plasma using high performance liquid chromatography. Eur J Drug Metab Pharmacokinet.

[22] Chappell CG, Creaser CS, Shepherd MJ (1992). Modified on-column interface for coupled high-performance liquid chromatography-gas chromatography and its application to the determination of levamisole in milk. J Chromatogr A.

[23] Abdalraheem A (2020). Stability-indicating high-performance liquid chromatographic determination of levamisole hydrochloride in bulk and dosage forms. J Chem.

[24] El-Kholy H, Kemppainen BW (2005). Levamisole residues in chicken tissues and eggs. Poult Sci.

[25] Tyrpenou AE, Xylouri-Frangiadaki EM (2006). Determination of levamisole in sheep muscle tissue by high-performance liquid chromatography and photo diode array detection. Chromatographia.

[26] Sowjanya S, Devadasu C (2018). Development of RP-HPLC method for the simultaneous quantitation of levamisole and albendazole: application to assay validation. Int J Anal Chem.

[27] Gao P (2021). Foods.

[28] Chen L (2022). Simultaneous determination of levamisole, mebendazole, and the two metabolite residues of mebendazole in poultry eggs by high-performance liquid chromatography-tandem mass spectrometry. Separations.

[29] Cherlet M (2000). Quantitative analysis of levamisole in porcine tissues by high-performance liquid chromatography combined with atmospheric pressure chemical ionization mass spectrometry. J Chromatogr B Biomed Sci Appl.

[30] Dreassi E (2001). Determination of levamisole in animal tissues using liquid chromatography with ultraviolet detection. J Agric Food Chem.

[31] Hess C (2014). Determination of levamisole, aminorex, and pemoline in plasma by means of liquid chromatography-mass spectrometry and application to a pharmacokinetic study of levamisole. Drug Test Anal.

[32] Cannavan A, Blanchflower WJ, Kennedy DG (1995). Determination of levamisole in animal tissues using liquid chromatography–thermospray mass spectrometry. Analyst.

[33] Gallo P, Fabbrocino S, Serpe L (2012). Determination of levamisole in feeds by liquid chromatography coupled to electrospray mass spectrometry on an ion trap. Rapid Commun Mass Spectrom.

[34] Jedziniak P, Szprengier-Juszkiewicz T, Olejnik M (2009). Determination of benzimidazoles and levamisole residues in milk by liquid chromatography–mass spectrometry: Screening method development and validation. J Chromatogr A.

[35] Santhakumari K. and Rao SMKP, Validated Method Development of Levamisole and Inosine pranobex by using UPLC &Characterization of Degradants by LC-MS/MS*.*

[36] Trehy ML (2011). Determination of levamisole in urine by gas chromatography-mass spectrometry. J Anal Toxicol.

[37] Chappell CG (1992). On-line high-performance liquid chromatographic/gas chromatographic/tandem ion trap mass spectrometric determination of levamisole in milk. Biol Mass Spectrom.

[38] Ahmed AMA (2022). Simultaneous determination of levamisole and oxyclozanide in the pharmaceutical preparation by capillary electrophoresis. Al-Azhar J Pharmaceutical Sci.

[39] Sharma R, Kumar J (2018). Development and validation of a high-performance thin-layer chromatographic method for the simultaneous determination of levamisole and cocaine in seized cocaine sample. JPC J Planar Chromatogr Modern TLC.

[40] Xu L (2013). Development and validation of a non-aqueous capillary electrophoresis method for simultaneous estimation of mebendazole and levamisole hydrochloride in compound mebendazole tablets. Anal Methods.

[41] Xu L (2014). Development of a capillary zone electrophoresis method for determination of mebendazole and levamisole hydrochloride in a combined tablet and a comparison with a LC method. J AOAC Int.

[42] El-Didamony AM (2008). Spectrophotometric determination of benzydamine HCl, levamisole HCl and mebeverine HCl through ion-pair complex formation with methyl orange. Spectrochim Acta Part A Mol Biomol Spectrosc.

[43] Sadeghi S, Fathi F, Abbasifar J (2007). Potentiometric sensing of levamisole hydrochloride based on molecularly imprinted polymer. Sens Actuators, B Chem.

[44] Khalil S, Borham N (2000). Indirect atomic absorption spectrometric determination of pindolol, propranolol and levamisole hydrochlorides based on formation of ion-associates with ammonium reineckate and sodium cobaltinitrite. J Pharm Biomed Anal.

[45] Xiao Y, Li J, Fu C (2014). A sensitive method for the determination of levamisole in serum by electrochemiluminescence. Luminescence.

[46] Seidi S (2011). Electromembrane extraction of levamisole from human biological fluids. J Sep Sci.

[47] Lourencao BC (2016). Amperometric flow-injection determination of the anthelmintic drugs ivermectin and levamisole using electrochemically pretreated boron-doped diamond electrodes. Sens Actuators, B Chem.

[48] Takeba K (2000). Simultaneous determination of triclabendazole and its sulphoxide and sulphone metabolites in bovine milk by high-performance liquid chromatography. J Chromatogr A.

[49] Rashed NS (2020). Development and validation of a green HPLC method for the analysis of clorsulon, albendazole, triclabendazole and ivermectin using monolithic column: Assessment of the greenness of the proposed method. Microchem J.

[50] Shurbaji M (2010). Development and validation of a New HPLC-UV method for the simultaneous determination of triclabendazole and ivermectin B1a in a pharmaceutical formulation. J AOAC Int.

[51] Ferretti R (2017). Enantiomers of triclabendazole sulfoxide: Analytical and semipreparative HPLC separation, absolute configuration assignment, and transformation into sodium salt. J Pharm Biomed Anal.

[52] Negro A (1992). Reversed-phase ion-pair high-performance liquid chromatographic determination of triclabendazole metabolites in serum and urine. J Chromatogr.

[53] Bull MS, Shume GRE (1987). A rapid high-performance liquid chromatographic procedure for the determination of triclabendazole and its metabolites in sheep plasma. J Pharm Biomed Anal.

[54] Alvinerie M, Galtier P (1986). Assay of triclabendazole and its main metabolites in plasma by high-performance liquid chromatography. J Chromatogr B Biomed Sci Appl.

[55] Asadi M, Haji Shabani AM, Dadfarnia S (2016). Simultaneous extraction and quantification of albendazole and triclabendazole using vortex-assisted hollow-fiber liquid-phase microextraction combined with high-performance liquid chromatography. J Sep Sci.

[56] Belal F (2014). Application of liquid chromatographic method with fluorescence detection for the determination of triclabendazole in tablets and biological fluids. Luminescence.

[57] Cai C (2010). Simultaneous determination of triclabendazole and its metabolites in bovine and goat tissues by liquid chromatography-tandem mass spectrometry. J Chromatogr B Analyt Technol Biomed Life Sci.

[58] Kikuchi H (2019). Total determination of triclabendazole and its metabolites in bovine tissues using liquid chromatography-tandem mass spectrometry. J Chromatogr B.

[59] Belal FF (2014). Stability-indicating spectroflurometric method for determination of triclabendazole in pure form and tablets. Anal Methods.

[60] Shrivastava A, Kumar S, Jain A (2011). Spectrophotometric method for quantitative determination of triclabendazole in bulk and pharmaceutical. Chronicles of Young Scientists.

[61] Pektas G, Dinç E, Baleanu D (2008). Spectrophotometric simultaneous determination of levamisole and triclabendazole in tablets by principal component regression and partial least squares chemometric methods. Revista de chimie-bucharest-original edition-.

[62] ICH, I. Q2 (R1): Validation of analytical procedures: text and methodology. in International conference on harmonization, Geneva. 2005.

[63] Wojnowski W (2022). AGREEprep—analytical greenness metric for sample preparation. TrAC, Trends Anal Chem.

[64] Pena-Pereira F, Wojnowski W, Tobiszewski M (2020). AGREE—analytical GREEnness metric approach and software. Anal Chem.

[65] Pena-Pereira F (2022). A tutorial on AGREEprep an analytical greenness metric for sample preparation. Advances in Sample Preparation.

[66] Płotka-Wasylka J, Wojnowski W (2021). Complementary green analytical procedure index (ComplexGAPI) and software. Green Chem.

[67] Płotka-Wasylka J (2018). A new tool for the evaluation of the analytical procedure: green analytical procedure index. Talanta.

[68] B. Magnusson and U. Örnemark (eds.) Eurachem Guide: The Fitness for Purpose of Analytical Methods – A Laboratory Guide to Method Validation and Related Topics. (2nd ed. 2014). ISBN 978–91–87461–59–0. http://www.eurachem.org.

[69] Food and D. Administration, Reviewer Guidance: Validation of chromatographic methods. Center for Drug Evaluation and Research. November, 1994.

[70] Sherma J, Fried B (2003). Handbook of thin-layer chromatography.

[71] Hagag MG (2023). Spectrum subtraction as a complementary method for six resolution techniques resolving overlapping spectra; application to multicomponent veterinary formulation with greenness and whiteness assessment. BMC Chemistry.

[72] De Ruyck H (2002). Development and validation of a liquid chromatographic–electrospray tandem mass spectrometric multiresidue method for anthelmintics in milk. J Chromatogr A.

[73] Dinç E, Baleanu D, Köktaş NŞ (2011). New spectral approaches to the simultaneous quantitative resolution of a combined veterinary formulation by ANN and PCA-ANN methods. Rev Anal Chem.

[74] Armitage, P., Further Experimental Designs: Statistical Methods in Medical Research. Edited by: Armitage P, Berry G. 1994, London: Blackwell Scientific Publications.

[75] Syed SM (2020). A Validated UV spectroscopic method for determination of levamisole HCl. Int J Pharm Res Appl.

